# Evaluation of a 13-Month-Old Anemic Child With Gushing Gums

**DOI:** 10.7759/cureus.42665

**Published:** 2023-07-29

**Authors:** Meghan E Guinee, Harsha Bhagtani

**Affiliations:** 1 Pediatrics, Edward Via College of Osteopathic Medicine, Blacksburg, USA

**Keywords:** bleeding gums, preventative health, gingivitis, oral hygiene, anemia in pediatrics

## Abstract

While it is common practice for adults to brush their teeth twice a day and instill in their children the importance of setting hygiene routines centered around brushing their teeth, infants and toddlers are often overlooked. Infants begin teething around four to seven months of age; during this period of tooth eruption, their gums are highly susceptible to bacterial build-up, causing problems long before oral hygiene comes to mind. We describe a case of a 13-month-old child presenting with bleeding gums and worsening anemia. After blood tests, iron supplementation, and a referral to a pediatric hematologist-oncologist out of concern for a potential bleeding disorder, this patient was diagnosed with normochromic, normocytic anemia caused by bleeding due to infectious gingivitis. After the completion of antibiotic therapy and changes to the patient's routine to incorporate appropriate oral hygiene, the anemia resolved. Generally benign, gingivitis induced by plaque biofilm can advance to more severe forms of periodontal disease, leading to receding gums and abscesses, thus reinforcing the importance of promoting adequate oral hygiene in all ages regardless of dentition. Additionally, educating primary-care providers on pediatric gingivitis allows for the inclusion of this diagnosis on differentials, limiting extensive blood tests and specialist appointments.

## Introduction

Gingivitis is an inflammation involving the tissue next to the tooth, or ‘gums’, and is caused by the build-up of bacterial plaque or soft bacterial deposits [1]. These bacterial plaque form from undisturbed film on teeth followed by bacterial adhesion which causes an imbalance within the diverse microbial community of the mucous membranes [2]. Under normal circumstances, the human mouth is home to a varied community of bacteria of over 700 different species [3]. When in harmonic balance, this arrangement benefits both organisms. However, when the parasitic burden overwhelms the host, it can lead to gingivitis and periodontal disease. Some common pathogenic bacteria responsible for gingivitis and other periodontal diseases include gram-negative bacteria and anaerobes such as *Actinobacillus sp.*, *Porphyromonas gingivalis*, and the spirochaete *Treponema denticola* [4]. However, infection is not the only cause of gingivitis and other conditions such as vitamin deficiencies should be evaluated when assessing a patient presenting with bleeding gums or other symptoms associated with gingivitis. Scurvy, a vitamin C deficiency, can cause spongy or sore bleeding gums, loose teeth, and delayed wound healing [5]. Vitamin K is essential to the clotting cascade; when the body is deficient, it can lead to bleeding [6]. While scurvy and vitamin K deficiencies are rare, screening remains important as simple interventions can resolve the disease. 

Gingivitis causes the gingiva to become red and swollen, usually becoming clinically symptomatic when the patient bleeds from teeth brushing and pressure on the gums or when halitosis fails to resolve following adequate oral hygiene. Gingivitis can be diagnosed based on clinical presentation and visual examination without the necessity of lab tests or imaging [7].

In infants and toddlers, gingivitis is not uncommon and is referred to as eruptive gingivitis, or an inflammation of the gums as new teeth push through the surface. Additionally, the malalignment of new teeth increases the gingiva’s susceptibility to accumulation of plaque. A study that surveyed 299 children found that eruptive gingivitis was found in 13.2% of those six to 17 months of age, 33.9% of the 18- to 23-month group, and 38.5% of the 24- to 36-month-old children [1]. Teeth brushing in children during tooth eruption can be difficult and even unpleasant for both the child and parent contributing to the presence in this patient population [8]. However, the incidence of gingivitis tends to increase with age, peaking in children aged six to seven years, when permanent teeth start to erupt [1]. This age group is not only susceptible to gingivitis induced by plaque biofilm for the same reasons as infants and toddlers, but parents may give children more autonomy over brushing teeth so quality and occurrence may decline.

In a prospective study researchers found that among children aged two to 11 years old, 41% had dental caries [9]. Dental caries, or tooth decay, is a result of bacterial acids causing demineralization of teeth, a similar etiology to the inflammatory response caused by bacterial plaque build up seen in gingivitis. These statistics emphasize the importance of not only encouraging parents to brush their children’s teeth but to teach children how to develop a routine and properly maintain their oral hygiene. Research has determined that periodontal disease in adults often begins to develop in childhood through years of bacterial plaque build-up and inadequate dental hygiene. Thus, aggressive prevention measures with oral health promotion and education should be encouraged from a very young age [1].

The consequences of gingivitis go beyond damage in the oral cavity causing tooth decay and abscesses; bacteria from gums can enter the bloodstream and cause systemic inflammation, by releasing inflammatory mediators such as IL-1, Tumor necrosis factor (TNF)-α, and interleukin (IL)-6 which result in elevations of inflammatory markers such as C-reactive protein (CRP) and erythrocyte sedimentation rate (ESR) [10]. Progression to a systemic inflammatory process has the potential to cause the patient serious complications, such as atherosclerosis and other cardiovascular diseases. This most commonly occurs in adults and is preventable and reversible with appropriate dental hygiene. Oral hygiene is not the sole solution to these inflammatory conditions but can play a role in the severity of disease and should be considered when counseling patients [11].

## Case presentation

A 13-month-old Caucasian boy presented to an outpatient clinic for follow-up hemoglobin check with a history of recurrent episodes of bleeding gums. Hemoglobin measured in the office was 8.3 g/dL. During history taking patient’s mother reported that at his 12-month well-child check, he was diagnosed with mild anemia after an in-clinic hemoglobin of 10.4 g/dL. The patient was started on an infant multivitamin with iron 1 mL daily and his mother was educated to increase his intake of iron-rich foods and decrease whole milk consumption. The patient was compliant with the treatment plan, but his mother noticed heavy bleeding during teeth brushing. At a scheduled dental appointment, his bleeding gums prevented the completion of the examination. At the appointment, the patient’s mother denied changes in appetite, fatigue, sleep disturbances, joint swelling, abnormal breathing patterns, and irritability. The child was uncircumcised, growing appropriately, and meeting all developmental milestones. The patient was not on any prescription medications and had no family history of bleeding disorders.

On physical examination, the patient was afebrile with vital signs within the normal limit for a 13-month-old child. The patient was alert, smiling, and playful during the visit. On examination of the skin, no rashes, bruising, or petechiae were noted. Evaluation of eyes demonstrated intact extraocular movements, no discharge, and normal conjunctiva bilaterally with no signs of pallor. Examination of the patient’s lips was negative for fissures or ulceration. On inspection of the oral cavity, there were eight erupted deciduous teeth, including the upper and lower central and lateral incisors. Additionally, there was evidence of bilateral upper molar progression without full eruption. The patient’s gingiva was moist and without lesions, but there was evidence of plaque deposits on gingival margins and tissue was swollen, erythematous . The patient’s tongue, buccal mucosa, and palate were unremarkable. The patient’s nares were patent and clear, without drainage. Examination of the neck was negative for lymphadenopathy. The remainder of the patient’s exam was unremarkable.

Laboratory analysis revealed a stool heme occult that was negative for blood. A complete blood count (CBC) was significant for a red blood cell count of 3.62M/μL, hemoglobin of 10.1 g/dL, a hematocrit of 28.6%, a mean platelet volume of 9.1 fL, and an absolute lymphocyte count of 59 K/μL (Table 1). The results of the iron and coagulation studies were all in the normative range (Table 2,3). The results of the reticulocyte count were 0.8 %, and an absolute reticulocyte count of 0.032% (Table 4). No imaging studies were indicated for this patient. A peripheral blood smear demonstrated normochromic, normocytic red blood cells.

**Table 1:** CBC with differential CBC: Complete blood count; WBC: White blood cell count; RBC: Red blood cell count; MCV: Mean corpuscular volume; MCH: Mean corpuscular hemoglobin; MCHC: Mean corpuscular hemoglobin concentration; RDW: Red cell distribution width; MPV: Mean platelet volume

Parameter	Admission value	Reference range
WBC, K/μL	6.1	6.0-17.0
RBC, M/μL	3.62	3.7-5.3
Hemoglobin, g/dL	10.1	10.5-13.5
Hematocrit, %	28.6	33-39
MCV, fL	79.0	70-86
MCH, pg	27.9	27-34.6
MCHC,	35.3	33-37
RDW	12.0	11.5-14.5
Platelet Count	310	130-400
MPV	9.1	9.4-12.4
Seg	24	15-85
Lymph	59	30-95
Mono	15	3-85
Eos	2	1.65-4.65
Absolute Neut	1.5	1.5-8.5
Absolute Lymph	59	4.0-10.5
Absolute Mono	0.9	0.05-1.1
Absolute Eos	0.1	0-0.4

**Table 2:** Results of iron studies TIBC: Total iron binding capacity

Parameter	Admission value	Reference range
Ferritin, ng/mL	161.7	30-400
Iron, UG/DL	72	50-120
TIBC, UG/DL	256	255-450

**Table 3:** Results of coagulation studies PT: Prothrombin time; PTT: Partial thromboplastin time; INR: International normalized ratio

Parameter	Admission value	Reference range
PT	12.0	11.6-15.2
INR	1.0	0.9-1.1
PTT	34.7	24.0-37.0

**Table 4:** Results of reticulocyte count

Parameter	Admission value		Reference range
Retic, %		0.8	2.0-5.0
Absolute Retic, M/μL		0.032	0.080-0.280
Immature Retic Fraction, %		4.8	2.3-36.6

## Discussion

This patient experienced a substantial rate of blood loss through his gingiva which resulted in a normocytic, normochromic anemia. The patient’s perplexing presentation lead to a substantial differential diagnosis, requiring blood tests and expert consultations to rule out more significant diagnoses, such as bleeding disorders or other autoimmune diseases. While these diagnoses are uncommon they have significant morbidity associations and must be considered when a child presents with symptomology of excessive bleeding gums and worsening anemia despite iron supplementation. In uncircumcised anemic male infants with bleeding gums, bleeding disorders, such as hemophilia, von Willebrand disease (VWD), and acute lymphocytic leukemia (ALL), must be on every physician's differential. Hemophilia is caused by a lack of blood clotting factors, most frequently factor VIII, and can present with excessive bleeding while brushing teeth or during dental procedures, such as in this case [12]. Hemophilia was ruled out in this patient after the laboratory values on the CBC, iron panel, and coagulation tests were within the normative range. In a patient with hemophilia laboratory results would be significant for low hemoglobin and red blood cell count in addition to a prolonged activated partial thromboplastin time test (aPTT). VWD is the most common inherited bleeding disorder. It is caused by low, absent, or malformed von Willebrand factor [13]. VWD can present in early life with excess bleeding during circumcision or oral mucosal bleeding following dental procedures but is usually associated with strong family history and abnormalities in the CBC and coagulation tests. This patient lacked a family history of bleeding disorders and had no significant abnormal laboratory values. ALL can also present with swollen, bleeding gums but was ruled out in this case due to a lack of systemic symptoms such as fever, pallor, and weight loss [14].

After consultation with a local pediatric dentist, it was determined that inflammation of the gingiva by excessive bacterial build-up was the culprit for this patient’s bleeding and leukocytosis. The gingiva in children during tooth eruption has thicker epithelium and increased vascularity, which in addition to giving it a more reddish color can also lead to increased bleeding during times of inflammation, which explains the anemia present in this patient. While acute simple gingivitis generally does not require treatment, due to this patient's leukocytosis and bleeding it was recommended by the local pediatrician to start a course of amoxicillin. Liquid amoxicillin was prescribed at 25 mg/kg/day in divided doses every 12 hours for five days. Upon literature review, the recommendations for patients requiring systemic antibiotics for gingivitis includes Penicillin VK with metronidazole or amoxicillin-clavulanate, and, for patients with penicillin allergies, clindamycin can be used as well [15]. The patients symptoms resolved and after changes in oral hygiene, the gingival inflammation declined. Repeat labs were not ordered. With adequate counselling and implementation of recommendations, this child should avoid recurrences and have no lasting effects from this diagnosis. 

## Conclusions

This case demonstrates the importance of oral hygiene in young children to prevent subsequent consequences of gingivitis. In rural and underserved communities where pediatric dentists have a month-long waitlist and adult dentists do not see patients under the age of three, it is important for primary care providers to educate parents on the importance of oral hygiene, starting before tooth eruption and continuing to make it a daily routine for the rest of the patient’s life. This case identifies a seemingly unrelated consequence of a common disease and places emphasis on the importance of preventative measures. Recommendations from the American Academy of Pediatrics and the American Associated of Pediatric Dentistry both emphasize the importance of beginning good oral hygiene practices as soon as the baby is born, with gum wiping before tooth eruption and using fluorinated toothpaste and trips to the dentist once teething has begun.

## References

[1] McDonald RE, Avery DR, Weddell JA, John V (2011). Gingivitis and periodontal disease. McDonald and Avery Dentistry for the Child and Adolescent.

[2] Abusleme L, Hoare A, Hong BY, Diaz PI (2021). Microbial signatures of health, gingivitis, and periodontitis. Periodontol 2000.

[3] Paster BJ, Boches SK, Galvin JL (2001). Bacterial diversity in human subgingival plaque. J Bacteriol.

[4] Al-Ghutaimel H, Riba H, Al-Kahtani S, Al-Duhaimi S (2014). Common periodontal diseases of children and adolescents. Int J Dent.

[5] Van der Velden U (2020). Vitamin C and its role in periodontal diseases-the past and the present: a narrative review. Oral Health Prev Dent.

[6] Rajes E, Mangai TA, Babu NA, Malathi L (2020). Gingival bleeding - systemic causes. Eur J Mol Clin Med.

[7] Summers Summers, A A (2009). Gingivitis: diagnosis and treatment. Emergency Nurse.

[8] Bimstein E, Matsson L (1999). Growth and development considerations in the diagnosis of gingivitis and periodontitis in children. Pediatr Dent.

[9] Beltrán-Aguilar ED, Barker LK, Canto MT (2005). Surveillance for dental caries, dental sealants, tooth retention, edentulism, and enamel fluorosis--United States, 1988-1994 and 1999-2002. MMWR Surveill Summ.

[10] Eberhard J, Grote K, Luchtefeld M (2013). Experimental gingivitis induces systemic inflammatory markers in young healthy individuals: a single-subject interventional study. PLoS One.

[11] Panagakos F, Scannapieco Scannapieco, F F (2011). Periodontal inflammation: from gingivitis to systemic disease?. Gingival Diseases: Their Aetiology, Prevention and Treatment.

[12] Kulkarni R, Soucie JM (2011). Pediatric hemophilia: a review. Semin Thromb Hemost.

[13] Nichols WL, Hultin MB, James AH (2008). von Willebrand disease (VWD): evidence-based diagnosis and management guidelines, the National Heart, Lung, and Blood Institute (NHLBI) Expert Panel report (USA). Haemophilia.

[14] Valéra MC, Noirrit-Esclassan E, Pasquet M, Vaysse F (2015). Oral complications and dental care in children with acute lymphocytic leukemia. J Oral Pathol Med.

[15] Slots Slots, J J, Ting M (2002). Systemic antibiotic in the treatment of periodontal disease. Periodontol 2000.

